# Association between low vitamin D levels and the diagnosis of asthma in children: a systematic review of cohort studies

**DOI:** 10.1186/1710-1492-10-31

**Published:** 2014-06-11

**Authors:** Mhd Hashem Rajabbik, Tamara Lotfi, Lina Alkhaled, Munes Fares, Ghada El-Hajj Fuleihan, Salman Mroueh, Elie A Akl

**Affiliations:** 1 Clinical Research Institute, American University of Beirut, Beirut, Lebanon; 2 Department of Pediatrics and Adolescent Medicine, American University of Beirut, Beirut, Lebanon; 3 Department of Internal Medicine, Calcium Metabolism and Osteoporosis Program, WHO Collaborating Center for Metabolic Bone Disorders, American University of Beirut, Beirut, Lebanon; 4 Department of Clinical Epidemiology and Biostatistics, McMaster University, Hamilton, Ontario, Canada; 5 Department of Medicine, State University of New York at Buffalo, Buffalo, New York, USA

**Keywords:** Asthma, Wheezing, Childhood, Pediatric, Vitamin D, Bronchial hyper responsiveness, Lung function tests, Systematic review, Cohort

## Abstract

**Background:**

There is conflicting evidence about the association between low vitamin D levels in children and development of asthma in later life. The objective of this study was to systematically review the evidence for an epidemiological association between low serum levels of vitamin D and the diagnosis of asthma in children.

**Methods:**

We used the Cochrane methodology for conducting systematic reviews. The search strategy included an electronic search of MEDLINE and EMBASE in February 2013. Two reviewers completed, in duplicate and independently, study selection, data abstraction, and assessment of risk of bias.

**Results:**

Of 1081 identified citations, three cohort studies met eligibility criteria. Two studies found that low serum vitamin D level is associated with an increased risk of developing asthma late in childhood, while the third study found no association with either vitamin D2 or vitamin D3 levels. All three studies suffer from major methodological shortcomings that limit our confidence in their results.

**Conclusions:**

Available epidemiological evidence suggests a potential association between low serum levels of vitamin D and the diagnosis of asthma in children. High quality studies are needed to reliably answer the question of interest.

## Background

Asthma is a highly prevalent respiratory condition in childhood [1]. Development of asthma is associated with many immunological markers [2]. Hypovitaminosis is prevalent worldwide, and an increasing body of evidence supports pleotropic effects of vitamin D on various chronic disorders including those associated with immune regulatory function [3-5]. This includes associations with a number of childhood disorders [6], such as type I diabetes mellitus [7-11], celiac disease, and asthma [12,13].

The hypothesis is that vitamin D has immunoregulatory properties [14,15] that protect from asthma [16,17]. Vitamin D has been shown to play a role in both the innate and adaptive immune responses by promoting phagocytosis and modulating the effect of Th1, Th2 and regulatory T cells [18]. Vitamin D has also been shown to inhibit the production of TH17 cytokines, which are associated with the severity of asthma and low steroid responsiveness [19]. A number of studies have suggested that low vitamin D levels are associated with increased risk of developing asthma [20-22] but other studies failed to confirm these findings [23,24]. One cohort study has shown that vitamin D supplementation in childhood might be associated with an increased risk of developing asthma [25].

Given these conflicting results and the high prevalence of both asthma and vitamin D deficiency in many countries [26,27], we aimed to systematically review the evidence for the epidemiological association between low levels of serum vitamin D and asthma diagnosis in children.

## Methods

### Protocol and registration

Prior to starting the review process, we registered the systematic review protocol with PROSPERO (CRD42013004204) [28].

### Selection criteria

We included studies meeting the following eligibility criteria:

● *Types of studies*: cohort studies. We excluded case–control studies and cross sectional studies.

● *Types of participants*: children less than 18 years old and free of asthma at the time of inclusion in the cohort. We did not consider other kinds of allergic conditions.

● *Types of exposure*: serum vitamin D levels in the child. We excluded studies of vitamin D levels in the pregnant mother or in the cord blood at the time of delivery and studies of vitamin D intake or supplementation.

● *Types of outcome measures*: asthma diagnosed based on doctor’s diagnosis, questionnaires, or spirometry measures.

We did not exclude studies based on language or date of publication, but excluded meeting abstracts.

### Search strategy

The OVID interface was used to electronically search MEDLINE and EMBASE (from date of inception to February 2013). The search strategy was designed with the help of a medical librarian. The search combined terms for asthma, vitamin D, and pediatric age group. It used both free text words and medical subject heading. We did not use any search filter for study design. Additional file 1 provides the full details of the search strategy.

In addition, we searched the grey literature (theses and dissertations) and the abstracts and proceedings from the following scientific meetings: American Thoracic Society (ATS), American College of Chest Physicians (ACCP), Pediatric Academic Societies, European Respiratory Society, and European Society for Pediatric Research. We also reviewed the references lists of included studies and publications available in the authors’ libraries. We searched forward for papers citing our included papers (ISI Web of Science). Finally, we contacted the authors of included studies inquiring about potentially eligible studies that we might have missed.

### Selection of studies

Two reviewers (LA, MF) screened the titles and abstracts of identified citations for potential eligibility in duplicate and independently. We obtained the full text for citations judged as potentially eligible by at least one of the 2 reviewers. The two reviewers (LA, MF) then screened the full texts for eligibility, in duplicate, and independently.

### Data collection

Two reviewers abstracted data from included studies in duplicate and independently. A senior team member (EAA) provided oversight. For each included study, the following information was abstracted: type, funding, population characteristics, exposure, outcomes assessed and the statistical data.

For both study selection and data collection steps, the reviewers used a pilot tested and standardized screening form and detailed instructions and resolved disagreement by discussion. A senior team member (EAA) provided oversight.

### Assessment of risk of bias in included studies

The two reviewers assessed the risk of bias in each included study in duplicate and independently. They resolved disagreements by discussion or with the help of a third reviewer (EAA) who provided oversight. Risk of bias was assessed using the following criteria: [29].

● Failure to develop and apply appropriate eligibility criteria (e.g., selection of exposed and unexposed in cohort studies from different populations).

● Flawed measurement of exposure (i.e., serum vitamin D levels).

● Flawed measurement of outcome (i.e., asthma diagnosis).

● Failure to adequately control confounding variables (e.g., failure of accurate measurement of all known prognostic factors, failure to match for prognostic factors and/or adjustment in statistical analysis).

● Incomplete follow-up.

We graded each potential source of bias as high, low or unclear.

### Data analysis and synthesis

We used the kappa statistic to calculate the agreement between the two reviewers for the assessment of trial eligibility. We were not able to meta-analyze the results of the included studies, as two analyzed vitamin D as a continuous variable [13,24], while the third study analyzed it categorized into tertiles [12]. We report both unadjusted and adjusted odds ratios (ORs) where available.

## Results

### Description of study selection

**Figure 1:**
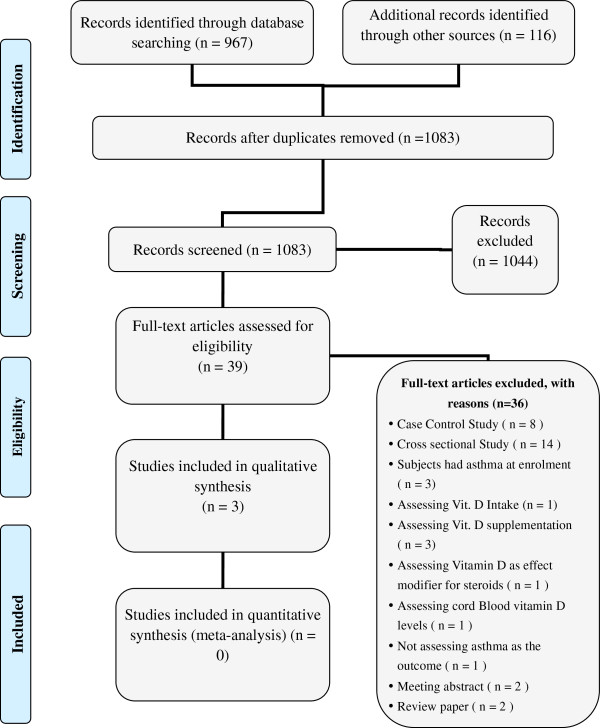
Study flow.

Figure 1 shows the study flow. Out of 39 potentially eligible studies, three met our eligibility criteria [12,13,24]. Additional file 2 lists the 36 excluded studies along with the reasons for exclusion.

### Study characteristics

Table 1 lists the characteristics of the included studies. All three studies used data collected in the context of larger cohort studies conducted in the late 1980’s and 1990’s. The first study was conducted in Australia and included a population of 989 six-year-old subjects followed up until 14 years of age. The second one was conducted in the Netherlands and included a population of 372 four-year-old subjects followed up until 8 years of age. The last study was conducted in England and included a population of 3,323 children with a mean age of 9.8 years and followed up until a mean age of 15.5 years.

**Table 1:** Characteristics of included studies

**Study name, Funding**	**Study design**	**Participants**	**Exposure**	**Outcome**	**Notes**
• Hollams [13]	• Prospective birth cohort started in 1989	• Conducted as part of West Australian Pregnancy Cohort (Raine Study): a longitudinal birth cohort, in which mothers (2900 volunteers) were enrolled for antenatal care at the main local tertiary maternity hospital	• Serum 25-hydroxyvitamin D levels measured at the age of 6 years	• Current asthma; defined as wheeze plus use of any asthma medication in the last 12 months, in children with a prior doctor diagnosis of asthma	• Vitamin D levels at age 6 years (Continuous outcome) analyzed as a predictor of subsequent clinical phenotypes at 14 years of age
Source of funding not reported					
	• Follow up: 8 years				
			• Measured using the enzyme immunoassay kit from Immunodiagnostic Systems Ltd (Scottsdale, AZ, USA)		
				• 8 years period between the point of measuring vitamin D levels and the assessment of asthma	• Reference group: sufficient level of vit D (> 75)
		• Included in this study: 989 children assessed at the age of 6 (no further details provided about selection criteria or process); 693 were included in the analysis.			
					• Vitamin D values were ‘deseasonalized’
				• Lung function; assessed by spirometry	
				• Bronchial hyperrsponsiveness (BHR); assessed by methacholine challenge.	
		• Perth, Western Australia, Australia			
				• Outcomes assessed at the age of 14 years	
Van Oeffelen [12]	• Prospective birth cohort started in 1996	• Conducted as part of PIAMA birth cohort of 3963 newborns; pregnant women recruited from the general population when visiting one of 52 prenatal clinics	• Serum 25-hydroxyvitamin D levels measured at the age of 4 years	• Asthma and severe asthma diagnosed using the (ISAAC) [29] questionnaire answered by parents annually until 8 year of age	• Vitamin D levels categorized into tetriles (Reference: tertile 1)
Funded by the Netherlands Organisation for Health Research and Development, the Netherlands Asthma Foundation, the Netherlands Ministry of Health, Welfare and Sport, and the National Institute of Public Health and the Environment.	• Follow up: 5 years				
					• Serum extracted and directly stored in a refrigerator at -20C, and defrosted in 2008 to measure of vit. D levels
			• Measured using a competitive enzyme immunoassay in microtiter plates (OCTEIA; IDS, Boldon, UK).		
				• 4 years period between the point of measuring vitamin D levels and the assessment of asthma	
		• Included in this study: 372 “selected” 4-year-old children (no further details provided about selection criteria or process); all were included in the analysis			
					• Storage time of serum samples proved to be no confounder and was therefore not added to the models.
			• Categorized into tertiles (range; median):		
				• Bronchial hyperrsponsiveness (BHR) measured at 8 years of age; assessed by methacholine challenge	
			o Tertile 1: 23.1–60.2; 52.0		
			o Tertile 2: 60.7–78.8; 68.3		• Vitamin D values were ‘deseasonalized’
		Netherlands			
			Tertile 3: 79.0–303.8; 97.0		
Tolppanen [22]	• Prospective birth cohort started in 1991	• Conducted as part of the Avon longitudinal Study of Parents and children (ALSPAC): 14,062 live births from 14,541 enrolled pregnant women who were expected to give birth between 1^st^ of April 1991 and 31^st^ of December 1992	• Serum 25-hydroxyvitamin D2 and D3 levels measured at a mean age of 9.8 years	• Asthma and wheezing assessed (questionnaire to children) at the age of 15-16 years.	• Asthma and wheezing also assessed on a yearly basis (questionnaire to caregiver); not clear whether those data were included in the analysis
Funded by the UK Medical Research Council, the Wellcome trust, and the University of Bristol					
	• Follow up: 6 years				
				• 5-6 years period between the point of measuring vitamin D levels and the assessment of asthma	
			• Measured using high pressure liquid chromatography tandem mass spectrometer in the multiple reaction mode		
					• The exposures are standardized for age and sex and 25(OH)D3 is adjusted for season and ethnicity
					• Interassay coefficients of
					• Variation for 25(OH)D2 and 25(OH)D3 were < 10% across a working range of 1–250 ng/ml
		• Lung function measured at a mean age of 15.5 years by spirometry according to the American Thoracic Society/European respiratory Society criteria. The best measurements from three reproducible flow-volume curves were used for analyses			
				• Included in this study (mean age of 9.8): 3323 children for the asthma outcome and 2,259 for spirometry (inclusion based on completeness of data on exposures, confounders, and outcome)	
		• South West England			

### Risk of bias

Table 2 describes the assessment of the risk of bias in those studies. In our judgment, the risk of bias associated with subject selection in the study by Van Oeffelen et al. was high as only a small percentage of the inception cohort was assessed in this study. The risk of bias associated with measurement of the exposure (Vitamin D levels) in the Van Oeffelen study was high due to the use of tests for which no validation is described, and due to the low reliability of a single measurement. The risk of bias associated with the outcome measurement (asthma) was low for both the Hollams et al. and Van Oeffelen et al. studies and uncertain for the Tolppanen et al. study in which non-validated questionnaires were used to diagnose asthma. We judged the risk of bias associated with confounding as high in one study due to lack of adjustment (Hollmans et al.). The risk of bias associated with missing data was judged as high in two studies due to high rates of missing data (30% for Hollams et al. and 42% for Tolppanen et al.).

**Table 2:** **Risk of bias in included studies**; **each criterion was graded as high**, **low**, **or unclear risk**

**Study name**	**Developing and applying appropriate eligibility criteria**	**Measurement of exposure**	**Measurement of outcome**	**Controlling for confounding**	**Completeness of data**
• Hollams [13]	• Uncertain risk	• High risk	• Low risk	• High risk	• High Risk
	• Although the risk of bias is low for the original cohort, no further details were provided about selection criteria or process for participants in this current study	• “Vit D levels was measured in thawed serum cryobanked at age 6 years” (number of years since blood draw not mentioned)	• Low for lung function and BHR	• Did not match or adjust for maternal atopy, maternal asthma, maternal age, education or household smoking	• Outcome data were missing for 30% of the enrolled cohort
		• The used enzyme immunoassay kit “method appeared to overestimate the vitamin D levels at age 6 years”			
		• Measuring Vitamin D levels at one point only may not be a reliable measure of integrated 25(OH) D levels over time			
Van Oeffelen [12]	• High risk	• High risk	• Low risk	• Low risk	• Uncertain Risk
	• Out of the larger cohort, a small “selected” sample included in this study, with no further details about selection provided	• Serum samples were defrosted to measure concentrations of Vitamin D (number of years since blood draw not mentioned)	for asthma (ISAAC score) Low risk for BHR	• Confounders were added to all models (gender, maternal atopy, paternal atopy, smoking by anyone in the house, and serum magnesium)	• Outcome data were missing for 12% of the enrolled cohort
		• Measured using a competitive enzyme immunoassay in microtiter plates			
		• Measuring Vitamin D levels at one point only may not be a reliable measure of integrated 25(OH) D levels over time		• Also considered playing outside and overweight as potential confounders	
Tolppanen [22]	• Uncertain risk	• High risk	• Uncertain risk for asthma, using spirometry & bronchidilatory responsiveness with non-validated questionnaires to diagnose asthma	• Low risk	• High risk
	• Except for the loss of follow up, the cohort was from a single community and followed specific eligibility criteria	• The exposures are standardized for age and sex and 25-hydroxyvitamin D3 is adjusted for season and ethnicity		• Model 1 unadjusted	• Of 5765 participants in the assessment of the wheezing and asthma outcome 3323 where included (42% missing data)
		• Measured using high pressure liquid chromatography tandem mass spectrometer in the multiple reaction mode (number of years since blood draw not mentioned)	• High risk for wheezing	• Models 2 and 3 adjusted for respectively 8 and 9 potential confounders	
			• Recall bias		• And of 4488 participants in the spirometric assessment 2259 where included (50% missing data).
		• Measuring Vitamin D levels at one point only may not be a reliable measure of integrated 25(OH) D levels over time			• Proportion of incident wheezing, and asthma was higher among children excluded owing to missing data

### Statistical results

#### Asthma

Hollams et al. [13] found an unadjusted OR for the association between increasing vitamin D levels and risk of asthma of 0.11 (95% CI 0.02–0.84). Van Oeffelen et al. found an adjusted OR of 0.39 (95% CI 0.27–0.53) when comparing vitamin D tertile 2 to tertile 1 (the lowest tertile) and an adjusted OR of 0.45 (95% CI 0.32–0.57) when comparing tertile 3 to tertile 1 (see Table 2 for variables used for adjustment) [12]. Tolppanen et al. found no association between vitamin D3 and asthma with an adjusted OR of 1.02 (95% CI 0.93–1.12) and an unadjusted OR of 0.98 (95% CI 0.92–1.05) [24]. Similarly, they found no association between vitamin D2 and asthma with an adjusted OR of 0.89 (95% CI 0.78–1.02) and an unadjusted OR of 0.98 (95% CI 0.89–1.08) (see Table 2 for variables used for adjustment). Hollams et al. also assessed the association between increasing vitamin D levels and severe asthma and found an OR of 0.28 (95% CI 0.06–1.37).

### Spirometric outcomes

Both Hollams et al. and van Oeffelen et al. assessed bronchial hyperresponsiveness using the methacholine challenge test. Hollams et al. [13] found an unadjusted OR for the association between increasing vitamin D levels and bronchial hyperresponsiveness of 0.28 (95% CI 0.06–1.37). Van Oeffelen [12] found an adjusted OR of 0.72 (95% CI 0.39–1.35) when comparing vitamin D tertile 2 to tertile 1, and an adjusted OR of 0.66 (95% CI 0.35–1.25) when comparing tertile 3 to tertile 1 (see Table 2 for variables used for adjustment).

Tolppanen et al. assessed lung function through the bronchodilator response to 400 ug dose of salbutamol. They reported the results using “SD change in outcome per doubling of exposure” [24]. This refers to reporting a standardized measure of the lung function outcome (typically equivalent to the mean divided by standard deviation) for every “doubling of exposure”. The authors calculated the doubling of exposure after scaling the two forms of 25(OH) D by multiplying the beta coefficients from the regression models by log_e_. The highest SD change reached among all analytical models was 0.06, which is typically considered a small effect size.

## Discussion

### Summary of main results

In summary, our systematic review identified three cohort studies assessing the association between low vitamin D levels in children and the incidence of asthma. Two of the included studies reported results suggesting that lower levels of vitamin D during childhood are associated with later development of asthma [12,13]. The third one found no association with either vitamin D2 or D3 levels [24]. Besides the inconsistent results, all three included studies suffered from major methodological limitations that limit our confidence in their findings. They all suffered to different degrees from selection bias (e.g., unclear selection criteria), inadequate measurement of the outcome (e.g., non-validated questionnaires), confounding (e.g., inadequate adjustment for potential confounders such as maternal atopy), and incompleteness of outcome data 12%-50% of subjects in the three studies had missing data). The risk of bias associated with exposure measurement was common to the three studies. Indeed, and as detailed in Table 2, vitamin D was measured once, between 5–8 years depending on the study, before the assessment of the outcomes, thus limiting the reliability of the value obtained as a reflection of integrated vitamin D nutritional status over the follow-up time [30]. In addition, the methods of handling the samples (e.g., thawing procedure) may have affected the accuracy of the results. Our confidence in the findings is further decreased by the imprecision of the results as indicated by the wide confidence intervals that include the value of 1 for most odds ratios.

### Overall completeness and applicability of evidence

Autier et al. recently published a systematic review of “vitamin D status and ill health” [31]. Although the reviewers included prospective cohort studies, they did not capture any of the studies we included in our review. This makes the possibility that we missed eligible studies unlikely. We have excluded studies of cord blood vitamin D levels because cord blood in part reflects maternal exposure, and evidence of their correlation with actual vitamin D levels during childhood is lacking. Also, we only included cohort studies to minimize the risk of bias in the results of this review. However, we observed that studies we excluded for their study design, and described as “case–control”, were actually cross-sectional in nature. Indeed, these studies assessed both the exposure (vitamin D level) and the outcome (asthma) simultaneously at the time of inclusion into the study.

### Agreements and disagreements with other reviews

Recently, Theodoratou et al. published an umbrella review that included systematic reviews and meta-analyses of observational studies of vitamin D and multiple health outcomes [32]. They identified no published systematic review or meta-analysis assessing the asthma outcome. We identified two systematic reviews [33,34] and two narrative reviews related to our study [35,36]. Neither of the two systematic reviews identified the studies we included in our review. The systematic review by Nurmatov et al. included studies related to maternal levels of vitamin D as opposed to children’s levels’. Most of the studies systematic review by Zhang et al. were either studies of chronic obstructive pulmonary disease or non-cohort studies [34]. The one cohort study of asthma that they included measured cord blood levels [37]. The narrative review by Gupta et al. addressed a range of questions around vitamin D and asthma in children [35]. That review did not include any of the three studies we included. The narrative review by Hollmas [36] identified only one of the three included studies [13].

### Implications for practice

The finding of a possible association between lower vitamin D levels and incidence of asthma may imply that vitamin D supplementation may be effective in preventing asthma. However, the considerable uncertainty regarding the validity of this association precludes at this point any translation of the findings into clinical practice. Indeed, we have identified 12 ongoing trials posted on clinicaltrials.gov that are assessing the effects of vitamin D supplementation in children with asthma symptoms.

### Implications for research

Future studies should address the methodological limitations of the available evidence. This includes proper assessment of the exposure, by assessing vitamin D status by multiple measurements throughout the study period, and accounting for seasonal variation and other environmental factors. It also requires setting appropriate eligibility criteria, valid measurement of outcomes, controlling for all confounders, and minimizing missing data.

## Abbreviations

BHR: Bronchial hyper responsiveness; 25(OH) Vitamin D: 25-Hydroxy Vitamin D.

## Competing interests

The authors declare that they have no competing interests.

## Authors' contributions

SM, EAA and LA conceived of the study and participated in the design of the study. LA and MF participated in study selection process. MR and TL participated in the data collection process. EA, GEHF and SM participated in data analysis and interpretation of the study. MR and EA helped in drafting the manuscript. All authors reviewed, edited, and approved the final manuscript.

## Supplementary Material

Additional file 1 Detailed search strategies. Click here for file

Additional file 2 Excluded Studies. Click here for file
