# Supplementary material for: Association between low vitamin D levels and the diagnosis of asthma in children: a systematic review of cohort studies
Source: Allergy Asthma Clin Immunol. 2014 Jun 11;10(1):31. doi: 10.1186/1710-1492-10-31 (PMC4064110; doi:10.1186/1710-1492-10-31)
Supplement: Additional file 1 — Detailed search strategies. [file 1710-1492-10-31-S1.pdf]

Appendix 1:

- MEDLINE: [(exp vitamin d/ or exp cholecalciferol/ or exp hydroxycholecalciferols/ or exp calcifediol/ or exp dihydroxycholecalciferols/ or exp calcitriol/ or exp 24,25-dihydroxyvitamin d 3/ or exp ergocalciferols/ or exp dihydrotachysterol/ or exp 25-hydroxyvitamin d 2/)OR (vitamin D or vitamin D2 or Vitamin D3 or ergocalciferol* or alfacacidol* or alfa-cacidol* or calcitriol* or cholecalciferol* or calciol* or calciferol* or hydroxycholecalciferol* or hydroxy-cholecalciferol* or dihydroxycholecalciferol* or dihydroxy-cholecalciferol* or 24,25-dihydroxyvitamin d3 or 24,25-dihydroxy-vitamin d3 or dihydrotachysterol* or dihydro-tachysterol* or 25-hydroxyvitamin d2 or 25-hydroxy-vitamin d2 or vitamin D deficien*)OR(exp vitamin d deficiency/ or exp osteomalacia/ or exp rickets/)] AND[(exp asthma/ or exp bronchial hyperreactivity/ or exp respiratory hypersensitivity/ or exp alveolitis, extrinsic allergic/ or exp aspergillosis, allergic bronchopulmonary/)OR((bronch* or respirat*) adj3 (hyperreactiv* or hyper-reactiv* or hypersensitiv* or hyper-sensitiv*))OR((asthma* or wheezing*).mp.OR((reactiv* adj3 airway*).mp.OR(exp anti-asthmatic agents/ or exp bronchodilator agents/)OR(anti asthmatic* or anti-asthmatic* or bronchodilator* or broncho-dilator*).mp.] AND[(exp pediatrics/ or exp neonatology/ or exp perinatology/)OR(exp adolescent/ or exp child/ or exp child, preschool/ or exp infant/ or exp infant, newborn/ or exp infant, low birth weight/ or exp infant, small for gestational age/ or exp infant, very low birth weight/ or exp infant, postmature/ or exp infant, premature/ or exp infant, extremely premature/)OR(exp Infant, Newborn, Diseases/)OR(infant* or child* or adolescen* or pediatric* or paediatric* or newborn* or new-born* or baby or babies or neonat* neo-nat* or toddler* or preschool* or pre-school* or teenage* or teen-age*).mp.]

- EMBASE: [(exp vitamin d/ or exp cholecalciferol/ or exp hydroxycholecalciferols/ or exp calcifediol/ or exp dihydroxycholecalciferols/ or exp calcitriol/ or exp 24,25-dihydroxyvitamin d 3/ or exp ergocalciferols/ or exp dihydrotachysterol/ or exp 25-hydroxyvitamin d 2/ or exp vitamin d deficiency/ or exp osteomalacia/ or exp rickets/)OR (vitamin D or vitamin D2 or Vitamin D3 or ergocalciferol* or alfacacidol* or alfa-cacidol* or calcitriol* or cholecalciferol* or calciol* or calciferol* or hydroxycholecalciferol* or hydroxy-cholecalciferol* or dihydroxycholecalciferol* or dihydroxy-cholecalciferol* or 24,25-dihydroxyvitamin d3 or 24,25-dihydroxy-vitamin d3 or dihydrotachysterol* or dihydro-tachysterol* or 25-hydroxyvitamin d2 or 25-hydroxy-vitamin d2 or vitamin D deficien*)]AND[(exp asthma/ or exp bronchial hyperreactivity/ or exp respiratory hypersensitivity/ or exp alveolitis, extrinsic allergic/ or exp allergic bronchopulmonary/ or exp anti-asthmatic agents/ or exp bronchodilator agents/)OR((bronch* or respirat*) adj3 (hyperreactiv* or hyper-reactiv* or hypersensitiv* or hyper-sensitiv*))OR(asthma* or wheezing*)OR(reactiv* adj3 airway*)OR(anti asthmatic* or anti-asthmatic* or bronchodilator* or broncho-dilator*)]AND[(exp pediatrics/ or exp neonatology/ or exp perinatology/ or exp adolescent/ or exp child/ or exp child, preschool/ or exp infant/ or exp infant, newborn/ or exp infant, low birth weight/ or exp infant, small for gestational age/ or exp infant, very low birth weight/ or exp infant, postmature/ or exp infant, premature/ or exp infant, extremely premature/ or exp Infant, Newborn, Diseases/)OR(infant* or child* or adolescen* or pediatric* or paediatric* or newborn* or new-born* or baby or babies or neonat* neo-nat* or toddler* or preschool* or pre-school* or teenage* or teen-age*)]
