# Supplementary material for: Association between low vitamin D levels and the diagnosis of asthma in children: a systematic review of cohort studies
Source: Allergy Asthma Clin Immunol. 2014 Jun 11;10(1):31. doi: 10.1186/1710-1492-10-31 (PMC4064110; doi:10.1186/1710-1492-10-31)
Supplement: Additional file 2 — Excluded Studies. [file 1710-1492-10-31-S2.pdf]

Appendix 2:

Study	Reason for exclusion
Carraro 2013 [1]	Cross sectional
Yasunori 2013 [2]	Cross sectional
Maalmi 2012[3]	Case-Control
Robinson 2012 [4]	Cross sectional
Menon 2012 [5]	Case-control
Majak 2012 [6]	Subjects had asthma at enrollment
WU 2012 [7]	Assessing Vitamin D as effect modifier for steroides
Brehm 2012 [8]	Cross sectional
Goleva 2012 [9]	Case-Control
Bener 2012 [10]	Case-Control
Taylor-Black 2012 [11]	Meeting abstract
Alyasin 2011[12]	Cross sectional
Frieri 2011 [13]	Review paper

Rothers 2011 [14]	Assessing cord blood vitamin D levels
Ma 2011 [15]	Cross sectional
Gupta 2011 [16]	Case-Control
Ehlayel 2011[17]	Case-Control
Chinellato 2011 [18]	Cross sectional
Sharief 2011 [19]	Cross sectional
Arnedo-Pena 2011 [20]	Cross sectional
Hughes 2011 [21]	Case-Control
Chinellato 2011 [22]	Cross sectional
Bueso 2011[23]	Case-Control
Freishtat 2010 [24]	Cross sectional
Searing 2010 [25]	Cross sectional
Emmanouil 2010 [26]	Cross sectional
Ciaccio 2010 [27]	Review paper
Brehm 2010 [28]	Subject had asthma at enrolment
Bose 2010 [29]	Subjects had asthma at enrollment

Koestner 2010 [30]	Not assessing asthma as the outcome
Brehm 2009 [31]	Cross sectional
Back 2009 [32]	Assessing Vitamin D Intake
Skripak 2009 [33]	Meeting abstract
Marmsjo 2009 [34]	Assessing Vitamin D supplementation
Kull 2006 [35]	Assessing Vitamin D supplementation
Hypponen 2004 [36]	Assessing Vitamin D supplementation

References

1. Carraro S, Giordano G, Reniero F, Carpi D, Stocchero M, Sterk PJ, Baraldi E: **Asthma severity in childhood and metabolomic profiling of breath condensate.** *Allergy: European Journal of Allergy and Clinical Immunology* 2013, **68:**110-117.
2. Ito Y, Adachi Y, Yoshida K, Akasawa A: **No association between serum vitamin D status and the prevalence of allergic diseases in Japanese children.** *Int Arch Allergy Immunol* 2013, **160:**218-220.
3. Maalmi H, Berraies A, Tangour E, Ammar J, Abid H, Hamzaoui K, Hamzaoui A: **The impact of vitamin D deficiency on immune T cells in asthmatic children: A case-control study.** *Journal of Asthma and Allergy* 2012**:**11-19.
4. Robinson CL, Baumann LM, Gilman RH, Romero K, Combe JM, Cabrera L, Hansel NN, Barnes K, Gonzalvez G, Wise RA, Breysse PN, Checkley W: **The Peru urban versus rural asthma (PURA) study: Methods and baseline quality control data from a cross-sectional investigation into the prevalence, severity, genetics, immunology and environmental factors affecting asthma in adolescence in Peru.** *BMJ Open* 2012, **2**.
5. Menon J, Maranda L, Nwosu BU: **Serum 25-hydroxyvitamin D levels do not correlate with asthma severity in a case-controlled study of children and adolescents.** *J Pediatr Endocrinol* 2012, **25:**673-679.
6. Majak P, Jerzynska J, Smejda K, Stelmach I, Timler D, Stelmach W: **Correlation of vitamin D with Foxp3 induction and steroid-sparing effect of immunotherapy in asthmatic children.** *Ann Allergy Asthma Immunol* 2012, **109:**329-335.
7. Wu AC, Tantisira K, Li L, Fuhlbrigge AL, Weiss ST, Litonjua A: **Effect of vitamin D and inhaled corticosteroid treatment on lung function in children.** *American Journal of Respiratory and Critical Care Medicine* 2012, **186:**508-513.
8. Brehm JM, Acosta-Perez E, Klei L, Roeder K, Barmada M, Boutaoui N, Forno E, Kelly R, Paul K, Sylvia J, Litonjua AA, Cabana M, Alvarez M, Colon-Semidey A, Canino G, Celedon JC: **Vitamin D insufficiency and severe asthma exacerbations in Puerto Rican children.** *Am J Respir Crit Care Med* 2012, **186:**140-146.
9. Goleva E, Searing DA, Jackson LP, Richers BN, Leung DY: **Steroid requirements and immune associations with vitamin D are stronger in children than adults with asthma.** *J Allergy Clin Immunol* 2012, **129:**1243-1251.
10. Bener A, Ehlayel MS, Tulic MK, Hamid Q: **Vitamin D deficiency as a strong predictor of asthma in children.** *Int Arch Allergy Immunol* 2012, **157:**168-175.
11. Taylor-Black SA, Wang J: **Vitamin D deficiency as a strong predictor of asthma in children.** *Pediatrics* 2012, **130:**S6.
12. Alyasin S, Momen T, Kashef S, Alipour A, Amin R: **The relationship between serum 25 hydroxy vitamin D levels and asthma in children.** *Allergy, Asthma and Immunology Research* 2011, **3:**251-255.
13. Frieri M: **The role of vitamin D in asthmatic children.** *Current Allergy and Asthma Reports* 2011, **11:**1-3.
14. Rothers J, Wright AL, Stern DA, Halonen M, Camargo CA, Jr.: **Cord blood 25-hydroxyvitamin D levels are associated with aeroallergen sensitization in children from Tucson, Arizona.** *J Allergy Clin Immunol* 2011, **128:**1093-1099.e1091-1095.
15. Ma XL, Zhen YF: **[Serum levels of 25-(OH)D(3) and total IgE in children with asthma].** *Zhongguo Dang Dai Er Ke Za Zhi* 2011, **13:**551-553.

16. Gupta A, Sjoukes A, Richards D, Banya W, Hawrylowicz C, Bush A, Saglani S: **Relationship between serum vitamin D, disease severity, and airway remodeling in children with asthma.** *Am J Respir Crit Care Med* 2011, **184:**1342-1349.
17. Ehlayel MS, Bener A, Sabbah A: **Is high prevalence of vitamin D deficiency evidence for asthma and allergy risks?** *Eur Ann Allergy Clin Immunol* 2011, **43:**81-88.
18. Chinellato I, Piazza M, Sandri M, Peroni DG, Cardinale F, Piacentini GL, Boner AL: **Serum vitamin D levels and exercise-induced bronchoconstriction in children with asthma.** *Eur Respir J* 2011, **37:**1366-1370.
19. Sharief S, Jariwala S, Kumar J, Muntner P, Melamed ML: **Vitamin D levels and food and environmental allergies in the United States: results from the National Health and Nutrition Examination Survey 2005-2006.** *J Allergy Clin Immunol* 2011, **127:**1195-1202.
20. Arnedo-Pena A, Garcia-Marcos L, Fernandez-Espinar JF, Bercedo-Sanz A, Aguinaga-Ontoso I, Gonzalez-Diaz C, Carvajal-Uruena I, Busquet-Monge R, Suarez-Varela MM, de Andoin NG, et al: **Sunny hours and variations in the prevalence of asthma in schoolchildren according to the International Study of Asthma and Allergies (ISAAC) Phase III in Spain.** *Int J Biometeorol* 2011, **55:**423-434.
21. Hughes AM, Lucas RM, Ponsonby AL, Chapman C, Coulthard A, Dear K, Dwyer T, Kilpatrick TJ, McMichael AJ, Pender MP, et al: **The role of latitude, ultraviolet radiation exposure and vitamin D in childhood asthma and hayfever: an Australian multicenter study.** *Pediatr Allergy Immunol* 2011, **22:**327-333.
22. Chinellato I, Piazza M, Sandri M, Peroni D, Piacentini G, Boner AL: **Vitamin D serum levels and markers of asthma control in Italian children.** *J Pediatr* 2011, **158:**437-441.
23. Bueso AK, Berntsen S, Mowinckel P, Andersen LF, Lodrup Carlsen KC, Carlsen KH: **Dietary intake in adolescents with asthma--potential for improvement.** *Pediatr Allergy Immunol* 2011, **22:**19-24.
24. Freishtat RJ, Iqbal SF, Pillai DK, Klein CJ, Ryan LM, Benton AS, Teach SJ: **High prevalence of vitamin D deficiency among inner-city African American youth with asthma in Washington, DC.** *J Pediatr* 2010, **156:**948-952.
25. Searing DA, Zhang Y, Murphy JR, Hauk PJ, Goleva E, Leung DY: **Decreased serum vitamin D levels in children with asthma are associated with increased corticosteroid use.** *J Allergy Clin Immunol* 2010, **125:**995-1000.
26. Emmanouil E, Manios Y, Grammatikaki E, Kondaki K, Oikonomou E, Papadopoulos N, Vassilopoulou E: **Association of nutrient intake and wheeze or asthma in a Greek pre-school population.** *Pediatric Allergy and Immunology* 2010, **21:**90-95.
27. Ciaccio CE, Portnoy JM: **Vitamin D insufficiency and risk of severe asthma exacerbations.** *Current Allergy and Asthma Reports* 2010, **10:**387-388.
28. Brehm JM, Schuemann B, Fuhlbrigge AL, Hollis BW, Strunk RC, Zeiger RS, Weiss ST, Litonjua AA: **Serum vitamin D levels and severe asthma exacerbations in the Childhood Asthma Management Program study.** *Journal of Allergy and Clinical Immunology* 2010, **126:**52-58.e55.
29. Bose S, Breysse P, Curtin-Brosnan J, McCormack MC, Hansel NN, Rusher RR, Matsui E, Diette G: **Outdoor exposure and vitamin D levels in urban children with asthma.** *American Journal of Respiratory and Critical Care Medicine* 2010, **181 (1 MeetingAbstracts)**.
30. Koestner N, Hartert TV, Newman MS, Gebretsadik TS, Liu Z, Carroll K, Minton PA, Woodward KB, Larkin EK: **Measurement of vitamin d levels utilizing laboratory and dietary recall information from the tennessee children's respiratory initiative.**

American Journal of Respiratory and Critical Care Medicine 2010, **181 (1 MeetingAbstracts)**.

31. Brehm JM, Celedon JC, Soto-Quiros ME, Avila L, Hunninghake GM, Forno E, Laskey D, Sylvia JS, Hollis BW, Weiss ST, Litonjua AA: **Serum vitamin D levels and markers of severity of childhood asthma in Costa Rica.** *Am J Respir Crit Care Med* 2009, **179:**765-771.
32. Back O, Blomquist HK, Hernell O, Stenberg B: **Does vitamin D intake during infancy promote the development of atopic allergy?** *Acta Derm Venereol* 2009, **89:**28-32.
33. Skripak JM: **Serum vitamin D levels and markers of severity of childhood asthma in Costa Rica.** *Pediatrics* 2009, **124:**S139-S140.
34. Marmsjo K, Rosenlund H, Kull I, Hakansson N, Wickman M, Pershagen G, Bergstrom A: **Use of multivitamin supplements in relation to allergic disease in 8-y-old children.** *Am J Clin Nutr* 2009, **90:**1693-1698.
35. Kull I, Bergstrom A, Melen E, Lilja G, van Hage M, Pershagen G, Wickman M: **Early-life supplementation of vitamins A and D, in water-soluble form or in peanut oil, and allergic diseases during childhood.** *J Allergy Clin Immunol* 2006, **118:**1299-1304.
36. Hypponen E, Sovio U, Wjst M, Patel S, Pekkanen J, Hartikainen AL, Jarvelinb MR: **Infant vitamin d supplementation and allergic conditions in adulthood: northern Finland birth cohort 1966.** *Ann N Y Acad Sci* 2004, **1037:**84-95.
